# High-Capacity Adsorption of a Cationic Dye Using Alkali-Activated Geopolymers Derived from Agricultural Residues

**DOI:** 10.3390/ma19010177

**Published:** 2026-01-03

**Authors:** Claudia Alejandra Hernández-Escobar, América Susana Mares-García, Miguel Alonso Orozco-Alvarado, Alejandro Vega-Rios, Claudia Ivone Piñón-Balderrama, Anayansi Estrada-Monje, Erasto Armando Zaragoza-Contreras

**Affiliations:** 1 Centro de Investigación en Materiales Avanzados, S.C., Miguel de Cervantes No. 120, Complejo Industrial Chihuahua, Chihuahua 31136, México; america.mares@cimav.edu.mx (A.S.M.-G.); miguel.orozco@cimav.edu.mx (M.A.O.-A.); alejandro.vega@cimav.edu.mx (A.V.-R.); claudia.pinon@cimav.edu.mx (C.I.P.-B.); 2 Secretaría de Ciencia, Humanidades, Tecnología e Innovación-Centro de Investigación en Materiales Avanzados, SC, Miguel de Cervantes No. 120, Complejo Industrial Chihuahua, Chihuahua 31136, México; 3 Centro de Innovación Aplicada en Tecnologías Competitivas, C. Omega 201, Industrial Delta, León de los Aldama 37545, México; aestrada@ciatec.mx

**Keywords:** geopolymer, wheat husk ash, methylene blue, wastewater treatment, sustainable materials

## Abstract

A geopolymer, derived from agricultural waste, was used as an efficient, sustainable, and low-cost adsorbent of methylene blue, a recurrent industrial dye contaminant. The geopolymer was synthesized via a standard alkali activation process using wheat husk ash calcinated at 1050 °C. Adsorption capabilities were evaluated through batch kinetic experiments. The removal efficiency was determined by ultraviolet–visible spectrophotometry, and the adsorption kinetics were fitted to various models. The geopolymer demonstrated a maximum adsorption capacity of 270.58 mg/g for methylene blue, achieving a removal efficiency of 85.20% under optimal conditions. Kinetic analysis confirmed that the adsorption process is best described by the pseudo-second-order model. This suggests that chemisorption, which involves chemical bonding or electron exchange between the dye and the negatively charged aluminosilicate structure of the geopolymer, is the rate-limiting mechanism. This demonstrates that geopolymers are effective and promising adsorbents, valorizing an agricultural waste stream into a functional material for the efficient treatment of dye-polluted wastewater. The competitive capacity and favorable chemisorption mechanism position the geopolymer as a promising material for the remediation of dye-contaminated industrial effluents.

## 1. Introduction

Water pollution is major worldwide issue that directly affects public health and environmental sustainability. The World Health Organization estimates that inadequate access to safe drinking water and sanitation is responsible for approximately 829,000 deaths each year [1]. Among the principal contributors to water contamination are the textile and printing industries, which release large volume of colored wastewater into natural water bodies [2], despite various traditional water treatment techniques available, such as electrochemical osmosis, flocculation, coagulation, and precipitation [1]. These methods are often associated with high operational costs and the generation of hazardous chemical sludge [3].

In this context, adsorption has emerged as one of the most effective strategies for the removal of both organic and inorganic contaminants from polluted waters. It is favored due to its simplicity, high performance, and cost-effectiveness compared to other methods [1,4]. The literature reports various low-cost adsorbent substances, including fly ash, activated carbon, zeolites, geopolymers, and clays [1].

Growing interest in sustainable and efficient adsorbents has driven the development of geopolymers (GPs). These inorganic polymers are amorphous to semi-crystalline aluminosilicate materials produced by the alkali activation and polycondensation of silicon (Si) and aluminum (Al)-rich precursors [1,5,6]. Structurally, GPs consist of a negatively charged aluminosilicate network balanced by exchangeable alkali cations such as Na^+^, K^+^, or Cs^+^ [6,7]. This makes them intrinsically porous and highly feasible for use as adsorbents [6]. Importantly, these balanced cations can exchange with cationic pollutants, such as cationic dye methylene blue (MB), providing a direct mechanism for dye removal [7].

GPs have been extensively studied for the immobilization and removal of heavy metal ions, including Pb^2+^, Cu^2+^, Cd^2+^, Ni^2+^, and Cr^6+^ [8]. The predominant mechanisms for heavy metal removal include electrostatic attraction, ion exchange (mainly with Na^+^ or K^+^ in the matrix), and surface complexation with functional groups such as hydroxyl (–OH) and carboxyl (–COOH) [3]. In addition, GPs have shown strong affinity for cationic radionuclides such as Cs^+^, Sr^2+^, and Co^2+^, making them promising materials for radioactive waste management [9,10]. The effectiveness of GPs in adsorbing other inorganic contaminants such as ammonium (NH_4_^+^) and phosphate, as well as organic compounds such as formaldehyde and various antibiotics, has also been evaluated [9,11]. Adsorbent morphology plays a decisive role in performance, with powdered GP forms generally exhibiting higher adsorption capacities than monolithic structures due to their larger specific surface areas [9].

Studies have confirmed the effectiveness of GPs in removing MB from aqueous solutions [12,13]. Reported maximum adsorption capacities (*Q_max_*) vary significantly depending on material composition and structural characteristics [14,15]. Significant maximum capacities have been reported, such as 43.48 mg/g for a metakaolin (MK) GP, 64.10 mg/g for porous GPs derived from pyrophyllite clay, 80.65 mg/g for a nanoGP synthesized from fired brick waste, and even 103.19 mg/g for a titanium dioxide-modified fly ash GP [5,16,17,18]. Furthermore, the development of composites has achieved even higher capacities, with the phosphoric acid/activated carbon GP reaching up to 204.08 mg/g [19], highlighting the potential of these materials as low-cost alternatives to activated carbon [16]. The strong affinity of GPs for MB is attributed to the cationic nature of the dye and its electrostatic interaction with the negatively charged aluminosilicate framework [20]. This interaction is strongly influenced by the pH of the solution since an alkaline pH, in the range of 7–10 or higher, favors adsorption by increasing the concentration of OH^−^ and the negative charge on the adsorbent surface [13,17]. Additional mechanisms, including hydrogen bonding between MB nitrogen atoms and surface hydroxyl groups, as well as pore diffusion, contribute to the overall adsorption process [8].

Geopolymers are commonly synthesized from industrial byproducts or naturally occurring aluminosilicate sources, including metakaolin, coal fly ash, red mud, and blast-furnace slag [1,21]. More recently, agricultural residues have attracted attention as alternative precursors because of their wide availability and compatibility with circular-economy strategies [22]. Nevertheless, agricultural ashes are far from equivalent. Their performance during alkali activation is governed by factors such as amorphous phase content, mineralogical composition, alkali and alkaline-earth impurities, and thermal treatment history [23,24]. As a result, ashes derived from materials such as rice husk or sugarcane bagasse, despite their frequent use, may yield GPs with limited pore connectivity or heterogeneous gel structures due to incomplete dissolution or the persistence of crystalline silica phases [25,26].

The wheat husk ash (WHA) examined in this study originates from a native Northern Mexican wheat variety, exhibits a chemical and mineralogical profile that is particularly favorable for GP formation. After calcination, the ash is dominated by highly reactive amorphous silica (≈81 wt%), with comparatively low levels of residual crystalline phases and interfering impurities [27]. Such a composition enhances dissolution kinetics in alkaline media and promotes efficient polycondensation during geopolymerization [28]. Consequently, the resulting aluminosilicate network is structurally homogeneous and characterized by a high density of Si–O–Al linkages. This framework supports the development of a mesoporous architecture with interconnected nanometric pores and generates a large population of surface silanol and aluminol groups, which are critical for adsorption processes [27].

From an adsorption standpoint, these structural features are particularly advantageous. Under neutral to mildly alkaline conditions, surface oxygen groups partially deprotonate, producing negatively charged sites capable of interacting strongly with cationic species through electrostatic attraction [8]. In parallel, the presence of charge-balancing alkali cations within the geopolymeric matrix enables ion-exchange mechanisms to contribute to pollutant uptake [19,29]. Based on these considerations, GPs derived from the investigated WHA are expected to exhibit favorable characteristics for cationic dye adsorption arising from their precursor composition and resulting microstructure. In this study, MB is employed as a model dye to examine the adsorption behavior of the synthesized GP, with particular emphasis on adsorption capacity, kinetics, and their relationship to structural features developed during alkali activation. This work focuses on establishing a structure-adsorption relationship within a unique and well-defined system, thus contributing to a deeper understanding of how precursor reactivity influences the adsorption performance of waste-derived GPs.

## 2. Materials and Methods

### 2.1. Materials

The primary precursor materials were wheat husk, sourced from agricultural waste in Chihuahua State, Northern Mexico. The wheat husk ash, a highly pozzolanic white powder, was obtained by calcining the raw wheat husk at 1050 °C for 16 h. The calcined WHA was ground and passed through a 200-mesh sieve, yielding a powder with a particle size distribution between 75 and 148 µm.

The alkali-activating solutions were prepared using sodium hydroxide (NaOH, Sigma-Aldrich, 98%, St. Louis, MO, USA) at concentrations of 16 M; sodium silicate solution (Na_2_SiO_3_) (Golden Bell, Mexico City, Mexico) which contained about 27–29 wt% SiO_2_ and 8–9 wt% Na_2_O, giving an active solids content of approximately 37–38 wt% and a SiO_2_/Na_2_O mass ratio of ≈3.2, according to the supplier’s specifications, and distilled water (J.T. Baker, Phillipsburg, NJ, USA). For the adsorption experiments, the cationic dye MB (Golden Bell, product number 60100) was used. All solutions and cleaning procedures utilized 96% ethyl alcohol (CTR Scientific, Chihuahua, Mexico) and distilled water (J.T. Baker, Phillipsburg, NJ, USA). All reagents were used as delivered.

### 2.2. Geopolymer Synthesis

The GP used in this study was synthesized following the methodology established in our previous work [1,27]. Briefly, a predetermined mass of WHA was combined with the alkali-activating solution (NaOH + Na_2_SiO_3_). Homogenization was performed via mechanical stirring at 800 rpm for 3 min. The resulting paste was cast into cylindrical molds and subjected to hydraulic compression.

The materials were cured using a domestic microwave (KOR-6LYB, Daewoo, Seoul, Republic of Korea) for 3 min at 540 W. The final solid products were subsequently ground and sieved to obtain a uniform particle-size fraction before all adsorption tests. The microwave curing conditions were chosen based on prior work on the same WHA system [27], in which this power–time combination consistently yielded a well-developed amorphous aluminosilicate gel and mesoporous microstructure without inducing thermal cracking or excessive densification. These conditions were adopted to favor microstructural features relevant to adsorption performance, rather than maximizing compressive strength, which is not a relevant parameter for powdered adsorbents.

### 2.3. Characterization

The synthesized GPs were characterized using various techniques. Structural and morphological analyses included: Fourier-transform infrared spectroscopy (FTIR) was performed using a GX-FTIR (Perkin Elmer, Waltham, MA, USA) with an ATR accessory. Spectra were recorded over 4000–500 cm^−1^ with a resolution of 40 cm^−1^ (average of 30 scans). X-ray diffraction (XRD) was used to determine the crystalline structure using an X’Pert PRO RX04 (Malvern Panalytical, Almelo Overijssel, The Netherlands) with a scan range of 5 to 60°, a step size of 0.03330°, and a counting time of 60 s. Scanning electron microscopy (SEM) allowed us to observe the microstructure using a SU3500 (Hitachi, Chiyoda, Tokyo, Japan) operating at 10 kV and a working distance of 10 mm, utilizing secondary electron detectors. Thermogravimetric analysis (TGA) was used to study the thermal stability using an SDT Q600 (TA Instruments, New Castle, DE, USA) from room temperature to 800 °C under air at a heating rate of 10 °C/min. Ultraviolet–visible (UV-Vis) spectrophotometry permitted a quantitative analysis of MB concentration during kinetic studies using a Cary 5000 (Varian, Palo Alto, CA, USA).

### 2.4. Adsorption Experiments

#### 2.4.1. Preparation of Methylene Blue Solutions

A concentrated stock solution of MB (250 mg/L) was prepared by dissolving 0.250 g of MB powder in 1 L of distilled water. All working solutions used in the batch adsorption tests were prepared by diluting this stock solution to the desired initial concentrations (C_0_) using distilled water.

#### 2.4.2. Batch Adsorption Tests

The adsorption capacity of the GP was evaluated through batch experiments conducted at room temperature. Aliquots of 50 mL of MB solution at a fixed initial concentration of 250 mg L^−1^ were transferred into glass containers, and a precisely measured amount of WH-GP powder (0.05–0.50 g) was added to each container. Before each adsorption experiment, the pH of the MB solutions was adjusted to 7.0 ± 0.1 using dilute HCl or NaOH solutions (0.1 M) and measured with a calibrated pH-meter. All batch adsorption tests were carried out in triplicate under the same conditions, and the reported removal efficiencies and adsorption capacities correspond to the average values of three independent experiments.

The suspensions were stirred continuously at 250 rpm using a magnetic plate (Thermo Scientific, Waltham, MA, USA). Samples were withdrawn at predetermined contact times (2, 4, 6, and 8 h) to monitor the kinetics. After each time interval, the solid adsorbent was separated from the supernatant by filtration (Whatman No. 1 filter paper), and the residual MB concentration (*C_t_*) in the supernatant was determined by UV–Vis spectrophotometry at 665 nm.

For structural characterization after adsorption, a representative sample (WH-GP-MB) was prepared under the same batch conditions by mixing 35 mg of WH-GP with 50 mL of an aqueous MB solution at 250 mg L^−1^, stirring at room temperature for 6 h, and then separating the solid by filtration and drying it at room temperature for 24 h prior to XRD and FTIR analyses.

### 2.5. Adsorption Calculations and Kinetic Modeling

The percentage of MB removal (R%) and the amount of dye adsorbed at time t (*Q_t_*, in mg/g) were calculated using Equations (1) and (2):

(1) $$R\%=\frac{C_{0}-C_{t}}{C_{0}}\;\times 100$$

(2) $$Q_{t}=\frac{(C_{0}-C_{t})\times V}{M}$$

where *C*_0_ and *C_t_* (mg/L) are the initial and final (at time t) concentrations of the dye, respectively. *V* (L) is the volume of the solution, and *M* (g) is the mass of the adsorbent.

To investigate the adsorption mechanism and rate, the experimental data were fitted to the pseudo-first order (PFO) (Equation (3)) and pseudo-second order (PSO) (Equation (4)) kinetic models.

(3) $$\log\left(q_{e}-q_{t}\right)=\log\left(q_{e}\right)-\frac{k_{1}}{2.303}\;t$$

(4) $$\frac{t}{q_{t}}=\frac{1}{k_{2}q_{e}^{2}}+\frac{1}{q_{e}}\;t$$

where *q_e_* and *q_t_* (mg/g) are the adsorption capacities at equilibrium and at time *t*, respectively. *k*_1_ (h^−1^) and *k*_2_ (g/mg h) are the rate constants for the PFO and PSO models, respectively.

## 3. Results and Discussion

### 3.1. Adsorbent Characterization

#### 3.1.1. Structural Analysis and Morphological (XRD and SEM)

**Figure 1:**
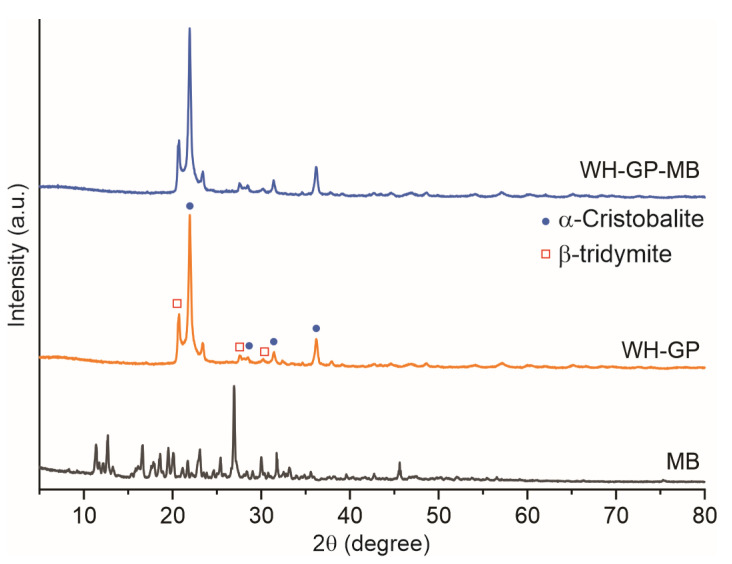
XRD pattern for WH-GP, WH-GP-MB, and MB. The α-cristobalite and β-tridymite phases were determined by Rietveld refinement using the FullProf software (2021 B cycle). The percentages obtained for each phase were 85.41% and 14.59%, respectively.

Figure 1 shows the XRD patterns of MB, WH-GP, and WH-GP-MB. XRD of MB powder shows several sharp crystalline reflections. The three most intense peaks in the measured pattern appear at ≈26.94°, 12.70°, and 11.38° 2θ, with additional weaker reflections in the 16–20° region. These reflections are consistent with reported X-ray powder patterns of MB hydrates/crystalline phases and indicate the dye is at least partly present in a crystalline (or highly ordered hydrate) form before adsorption [30].

The XRD pattern of the WH-GP sample is dominated by a broad diffuse halo between 20.7° and 36.2° (2θ), which is characteristic of an amorphous aluminosilicate GP gel [31,32]. The position and width of the amorphous hump are commonly associated with an advanced degree of geopolymerization and structural disorder, which are linked to a high density of negatively charged framework sites available for cationic species adsorption. This behavior is well documented for alkali-activated geopolymeric gels, confirming the formation of the amorphous aluminosilicate structure characteristic of GPs [28].

After MB adsorption (WH-GP-MB), the amorphous halo remains essentially unchanged, while the characteristic crystalline reflections of MB are no observed. This indicates successful dye immobilization without disruption of the GP matrix, suggesting that adsorption occurs predominantly via surface-related mechanisms such as electrostatic interaction and ion exchange rather than structural incorporation. This observation is consistent with other reports where dye adsorption onto amorphous aluminosilicate gels produces modest intensity changes (surface coating or pore-filling) but not the emergence of new crystalline reflections [16].

**Figure 2:**
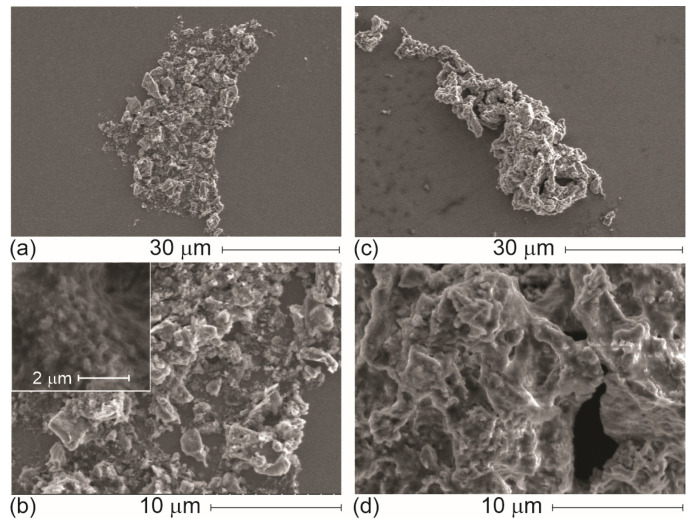
SEM images of WH-GP exposed to a solution of MB. (**a**,**b**) pristine WH-GP and (**c**,**d**) post-dye exposition.

Figure 2 depicts the morphological analysis of WH-GP before (Figure 2a,b) and after exposure to MB (Figure 2c,d). First, Figure 2a shows the microparticulate morphology of WH-GP, the product of mechanical milling and sieving with a 200-mesh screen (particle size distribution between 75 and 148 µm). As shown in this micrograph, the pristine WH-GP exhibits an irregular and heterogeneous morphology typical of alkali-activated systems, composed of agglomerated particles with rough, angular, and fractured surfaces [8]. The higher magnification (Figure 2b) highlights a textured surface characterized by flake-like features, microcracks, and interparticle voids. These morphological characteristics are indicative of an open microstructure with accessible surface sites with abundant accessible sites, appropriate for adsorption [17].

Following the adsorption process (WH-GP-MB), noticeable changes in surface morphology are observed. The GP particles appear more compact and locally smoother, with partial filling or masking of surface microstructure (Figure 2c). At higher magnification (Figure 2d), the previously sharp and irregular gel features are replaced by a more continuous surface with reduced apparent roughness. This morphological evolution suggests the deposition and retention of dye molecules within surface cavities and along GP gel domains, consistent with adsorption occurring at external surfaces and near-surface regions [3,17]. Despite these surface modifications, the overall particle integrity is preserved, and no evidence of structural collapse or cracking is observed after adsorption. This indicates that MB uptake does not compromise the stability of the GP matrix [14].

Importantly, WH-GP shows a rough surface microstructure with high porosity. The material’s physical texture and porous structure are directly responsible for generating and maintaining the surface area required for chemical and physical pollutant collection. A rough and porous microstructure is required because it significantly improves the specific surface area and accessibility of the internal structure to the liquid medium, allowing it to contact the pollutant [17]. Since MB is a cationic dye, the main interaction mechanism relies on “electrostatic attraction” between the positively charged dye cation and the “negative charge of the aluminosilicate backbone” (the geopolymeric network) [18]. This network consists of (SiO_4_) and (AlO_4_) groups linked by Si–O–Al– bonds. The negative charge is balanced by exchangeable cations (Na^+^ or K^+^) residing in the structural cavities. This electrostatic interaction is favored by alkaline pH (pH 7–10 or higher), which increases the concentration of hydroxyl ions (OH^−^) and subsequently, enhances the negative charge on the adsorbent surface [33].

The observed morphological changes therefore support an adsorption mechanism dominated by surface interaction and pore filling, rather than bulk structural transformation, in agreement with the XRD results.

The GP’s porosity was characterized previously [27]. N_2_ adsorption–desorption (BET/BJH) classified it as a mesoporous GP according to IUPAC criteria, with a BJH average pore diameter of 3.142 nm and a multi-point BET surface area of 0.7723 m^2^ g^−1^. This mesoporous aluminosilicate framework, with nanometric pores and a rough surface morphology observed by SEM, provides abundant and accessible silanol and aluminol sites for dye uptake, facilitating MB diffusion and anchoring within the pore network and helping to explain the relatively high maximum adsorption capacity (*Q_max_*) obtained in this study.

#### 3.1.2. Functional Group Analysis

**Figure 3:**
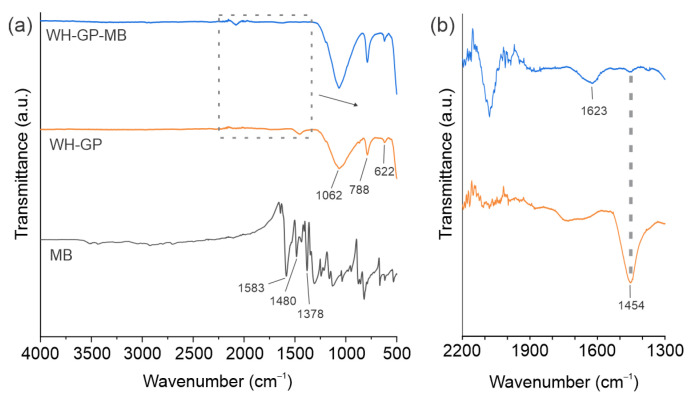
FTIR spectra of MB, WH-GP, and WHGP-MB. (**a**) WH-GP before and after dye adsorption and (**b**) amplification of the spectral region 1300–2200 cm^−1^. The gray dotted line and arrow indicate the expanded region of the spectrum.

Figure 3a shows FTIR spectra of MB, WH-GP, and WWH-GP-MB. For MB, the spectrum presents vibrations at 1378 cm^−1^ corresponding to the aromatic amine groups, several peaks in the range 1000–1250 cm^−1^, ascribed to the aliphatic amine. The adsorptions at 1480 cm^−1^ and 1583 cm^−1^ were respectively attributed to the heterocyclic moiety and the stretching of the aromatic ring vibrations [34]. The pristine WH-GP spectrum agrees with an alkali-activated aluminosilicate gel. The broad asymmetric Si–O–(Si/Al) stretching centered at 1062 cm^−1^, and the weaker bands at 788 and 616 cm^−1^, are attributed to Si–O–Si/Al bending and framework vibrations. A small band at 1454 cm^−1^ was ascribed to bound water [35]. After MB exposure, the WH-GP-MB spectrum retains the broad geopolymer Si–O band and shows a small absorption at 1623 cm^−1^, Figure 3b, attributed to bending of –OH bands [36]. This band is related to the main MB vibration identified and therefore indicates surface adsorption by MB [20]. As described in the previous section, the minor intensity changes after adsorption are consistent with weak interactions (electrostatic attraction, hydrogen bonding, and pore-filling) between MB^+^ and the geopolymeric network rather than structural transformation or breakdown of the aluminosilicate gel. Comparable observations have been reported in studies of MB adsorption onto aluminosilicate adsorbents and GPs and are commonly interpreted as dye deposition plus weak surface interactions rather than intercalation or chemical destruction of the host matrix [35].

### 3.2. Batch Adsorption Performance

**Figure 4:**
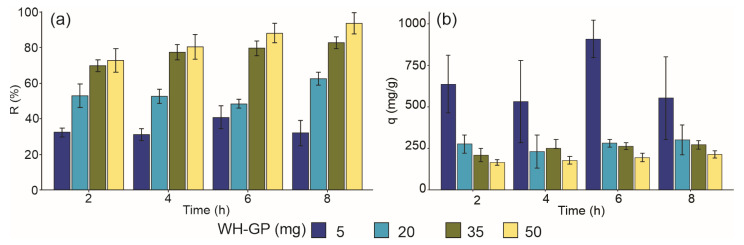
Adsorption properties of WH-GP for MB; (**a**) Effect of adsorbent dosage (WH-GP) on the MB removal efficiency (R%). Initial MB: 250 mg/L; contact time: 2, 4, 6, and 8 h. (**b**) Adsorption capacity (q) of WH-GP. Adsorbed capacity (q) in mg/g as a function of contact time: 2, 4, 6, and 8 h. Adsorbent dosage: 5, 20, 35, and 50 mg.

The batch adsorption experiments demonstrated that WH-GP exhibits a strong affinity for MB, with removal efficiency increasing proportionally to the adsorbent dosage (Figure 4a). This response is anticipated, as higher solid concentrations provide a greater number of accessible active sites and increased surface area. These active sites are principally the negatively charged surface groups (≡Si–O^−^ and ≡Al–O^−^) inherent to the aluminosilicate structure of WH-GP, which enhance the MB^+^ adsorption via electrostatic attraction [37]. However, increasing the adsorbent dosage beyond the optimal level may lead to saturation of available MB molecules or aggregation of the adsorbent particles, resulting in the adsorption capacity per unit mass decreasing even as the overall removal efficiency approaches a maximum [38]. This dosage-dependent enhancement in dye removal is a consistent trend reported for GPs, confirming that surface-site availability is a primary factor governing dye uptake in aluminosilicate-based adsorbents [18].

Figure 4a shows that increasing the WH-GP dosage from 5 to 50 mg (0.1–1.0 g L^−1^) led to a substantial enhancement in MB removal across all tested contact times. The removal efficiency improved from about 40% to more than 80%, reaching a maximum removal efficiency (R) of 85.20% after 8 h using 50 mg adsorbent dosage in 50 mL of stock solution (250 mg/L MB concentration), clearly demonstrating that WH-GP is capable of effectively removing high MB loads from aqueous solutions; this behavior can be attributed to the larger amount of adsorbent introduced into the system, which increases the number of accessible adsorption sites and facilitates a more extensive reduction of MB concentration in the aqueous phase.

In contrast, the adsorption capacity normalized to adsorbent mass (q) declined as the dosage increased, as illustrated in Figure 4b. This reduction is characteristic of a saturation phenomenon, in which once most MB molecules have been extracted from the solution; the excess adsorbent cannot be fully utilized, leaving part of the surface unoccupied. The inverse dependence of removal efficiency and specific adsorption capacity on adsorbent dose agrees with trends reported for GP- and aluminosilicate-based materials, emphasizing that the overall adsorption result is governed by the balance between available surface sites and the residual dye concentration in solution [39].

Regarding the kinetics, the adsorption progressed rapidly during the initial contact intervals (Figure 4b), which is typical for adsorption processes and is attributed to the high availability of vacant external surface sites and the strong concentration gradient acting as the initial driving force [13]. This rapid phase involves MB molecules interacting with external sites mainly through electrostatic attraction and ion exchange with charge-compensating cations (e.g., Na^+^) near the aluminosilicate framework [3], and it is often completed within the first 30–60 min [19]. As contact time increases, the slope of the q–t curves decrease, indicating that the most accessible high-energy sites are progressively saturated and that the system is approaching equilibrium. This slowdown is associated with the onset of slower mass-transfer mechanisms [18], such as intraparticle diffusion and pore transport within the internal mesopores and cavities of the WH-GP structure [37]. The rapid overall kinetics and the excellent pseudo-second-order fit indicate that the process is efficient and predominantly governed by strong chemical interactions (chemisorption).

### 3.3. Adsorption Kinetics

**Figure 5:**
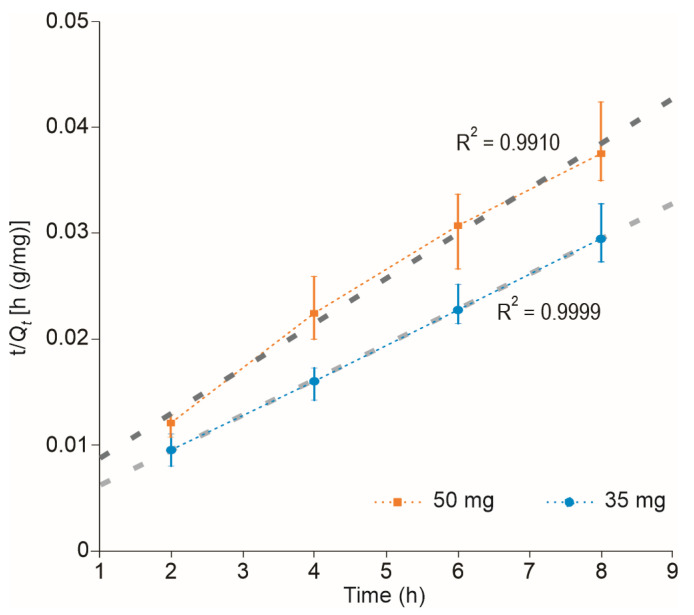
Kinetic modeling of MB adsorption onto WH-GP. Linear plot of the Pseudo-Second-Order model fit. The thin dotted lines indicates the joining of the experimental data and the thick dashed gray lines indicate the straight-line adjustments.

The adsorption kinetics of MB onto the WH-GP was evaluated using the PFO and PSO models. The kinetic analysis showed a coefficient of determination (R^2^) of 0.9999, with the PSO model, providing a substantially better fit than the PFO model (Figure 5). This determination was further supported by the closer agreement between the calculated and experimental equilibrium adsorption capacities [18]. This outcome is highly consistent with numerous studies on cationic dye adsorption onto GP and aluminosilicate-based adsorbents, which frequently confirm the PSO model as the best descriptor for the adsorption process [18,40]. The better fit to the PSO model indicates that the rate-limiting step is chemisorption. This implies that the removal is not controlled merely by simple mass transfer (physisorption) but involves specific surface interactions. Specifically, this mechanism involves electron sharing or exchange between the cationic MB^+^ and the negatively charged aluminosilicate sites (Si–O–Al) of the WH-GP structure. This phenomenon describes a two-step process commonly observed in these systems: an initial rapid electrostatic attraction, followed by a slower, stable anchoring phase characterized by chemisorption [41]. Although the PSO model suggests chemisorption dominates the rate, the resulting R^2^ value (0.9999) also implies that the overall adsorption mechanism is complex and multi-step, confirming that intraparticle diffusion and pore-transport phenomena may contribute during the later stages of adsorption [3,19].

The proposed mechanism is a two-step process:

1. Rapid initial electrostatic attraction: Under the alkaline conditions generated by NaOH/Na_2_SiO_3_, the WH-GP surface develops a high density of negative charges (=Si–O^−^) due to the deprotonation of oxygen groups. This promotes the rapid initial capture of MB^+^ ions, primarily driven by electrostatic attraction. This step is often accompanied by ion exchange, in which Na^+^ ions associated with the WH-GP surface are exchanged for MB^+^ ions as MB^+^ approaches. The Cl^−^ counterion of the dye does not participate in the anchoring and remains in solution.
2. Slower chemisorption step: In a second, slower stage crucial for the kinetics, the dye establishes stronger, specific interactions with the surface (e.g., hydrogen bonds with neighboring groups or coordinate bonding), giving this final stage a chemisorption character.

This rapid electrostatic affinity, followed by stable anchoring, is consistent with the excellent fit to the PSO model.

For the PSO model, the calculated equilibrium capacities (*q_e,cal_*) were 303.30 and 238.09 mg g^−1^ for adsorbent masses of 35 and 50 mg, respectively, while the experimental values (*q_e,exp_*) were 293.23 and 228.54 mg g^−1^. The close agreement between *q_e,cal_* and *q_e,exp_* confirms that the PSO model adequately describes the adsorption kinetics under the studied conditions (Table 1).

The wheat husk ash was obtained from a native Northern Mexican wheat variety, which exhibits a higher silica content (≈81 wt%) compared with other wheat varieties, (e.g., Turkish ≈ 58%, Indian ≈ 54%) [42,43], and pozzolanic reactivity, promoting the formation of a mesoporous aluminosilicate geopolymer framework with nanometric pores and abundant surface silanol and aluminol groups. Under neutral to alkaline conditions, these surface sites partially deprotonate to generate negatively charged Si–O^−^ and Al–O^−^ groups, providing electrostatically attractive and ion-exchange-active sites for cationic species such as MB.

The silica content, mesoporosity, negatively charged surface combined with the excellent fit to the pseudo-second-order model supports the high maximum adsorption capacity (*Q_max_* ≈ 270 mg g^−1^) obtained for WH-GP and confirm the potential and viability of this WHA-derived geopolymer as a high-capacity adsorbent for environmental remediation.

### 3.4. Comparative Adsorption Capacity

The experimental *q_e_* of 293.23 mg/g of WH-GP is a notable result. To contextualize this performance, a comparison with other GP-based adsorbents reported in the literature is presented in Table 2. Most conventional alkali-activated GPs show modest mg/g capacities depending on precursor and measurement conditions. For example, metakaolin-based geopolymers report *Q_max_* values in the 20–50 mg/g range, while simple biomass- or fly-ash-derived GPs give lower capacities [18]. On the other hand, GP composites and highly engineered hybrids tend to reach substantially higher *Q_max_* values (e.g., 80–204 mg/g or ~103 mg/g for a TiO_2_-modified fly-ash GPs), consistent with the idea that additional porosity and adsorptive phases strongly enhance adsorption [16,19].

Some factors may explain the WH-GP’s adsorption capacity: (i) the starting WHA used here is exceptionally silica-rich and, after calcination, produces highly reactive silica that can form a geopolymeric gel with a large density of silanol/aluminol surface sites and mesoporosity; (ii) microwave-assisted curing can accelerate gel formation and favors micro/mesopore architectures that improve accessibility and binding density; and (iii) the adsorption experiments fits can lead to higher numerical maxima when experiments include very high initial dye concentrations or when modified materials present strong chemisorptive interactions [16].

Because *Q_max_* values are very sensitive to experimental conditions (initial concentration range, temperature, pH, etc.) [12,33], the comparison should be taken as context rather than proof that one material is superior. Nonetheless, the WH-GP adsorption is among the highest reported geopolymer-based MB adsorbents, supporting the claim that WH-derived GPs are promising for high-capacity dye remediation.

## 4. Conclusions

In this study, WHA, an abundant agricultural residue, was valorized into a geopolymer-based adsorbent for MB removal from aqueous solutions. Under the experimental conditions evaluated, WH-GP exhibited a *Q_max_* of 270.58 mg/g and achieved a dye removal efficiency of 85.20% using a 50 mg adsorbent dosage in 50 mL of solution with an initial MB concentration of 250 mg/L over an 8 h contact time. This demonstrated a higher capacity than that of other GPs reported in the literature, positioning it as a competitive material. The adsorption behavior of MB onto WH-GP was analyzed using kinetic models, with the experimental data showing a notable fit to the pseudo-second-order model (R^2^ = 0.9999). This behavior suggests that adsorption is governed by interactions between the dye molecules and the active sites of the geopolymer surface, consistent with chemisorption-dominated processes commonly reported for geopolymer-based adsorbents. Within the scope of this study, the results evidenced that WHA from a native Northern Mexican source can be converted into a GP with effective adsorption performance toward MB. These findings support the feasibility of agricultural waste-derived GPs as adsorbent materials and provide a basis for future investigations under more complex scenarios.

## Figures and Tables

**Table 1:** Derived parameters for adsorption kinetics under various conditions.

Method (w AM (mg))	*k*_2_ × 10^−3^ (g/(h mg))	*q_e,cal_* (mg/g)	*q_e,exp_* (mg/g)	Removal (%)
PSO (35)	3.8893	303.30	293.23	75.76
PSO (50)	3.9200	238.09	228.54	85.20

PSO: Pseudo-second order.

**Table 2:** Comparison of maximum adsorbent capacity (*Q_max_*) of geopolymers for uptake MB.

Adsorbent	Precursor/Source	Time	*Q_max_* (mg/g)	Reference
Geopolymer (WH-GP)	Wheat Husk Ash	8 h	293.23	This study
Phosphoric-acid geopolymer + activated carbon (ACP)	Phosphoric-acid geopolymer foam + activated carbon composite	240 min	204.08	[19]
TiO_2_-modified fly-ash geopolymer	Fly ash + TiO_2_ nanoparticles	-	103.19	[17]
Nanogeopolymer	Fired-brick/burnt clay brick waste	~163 min	80.65	[16]
Fly ash geopolymer	Coal Fly Ash	75 h	18.3	[44]
Metakaolin-Based Geopolymer	Kaolin	3 h	43.48	[18]
Red Mud and Rice Husk Ash-Based Geopolymer Composites	Red Mud and Rice Husk Ash	3 h	3.9	[45]

## Data Availability

The original contributions presented in this study are included in the article. Further inquiries can be directed to the corresponding authors.
